# Management of Advanced Ovarian Cancer: Current Clinical Practice and Future Perspectives

**DOI:** 10.3390/biomedicines13071525

**Published:** 2025-06-22

**Authors:** Dimitrios Papageorgiou, Galateia Liouta, Evangelia Pliakou, Eleftherios Zachariou, Ioakeim Sapantzoglou, Ioannis Prokopakis, Emmanuel N. Kontomanolis

**Affiliations:** 1 Department of Gynecology, Athens Naval and Veterans Hospital, 11521 Athens, Greece; 2 Department of Medical Oncology, General Oncology Hospital of Kifissia “Agioi Anargiroi”, 14564 Athens, Greece; 3 Department of Medical Oncology, Athens Naval and Veterans Hospital, 11521 Athens, Greece; 4 1st Gynecology Department, Metropolitan General Hospital, 15562 Athens, Greece; 5 1st Department of Obstetrics and Gynecology, National and Kapodistrian University of Athens, 11527 Athens, Greece; 6 Department of Obstetrics and Gynecology, Democritus University of Thrace, 68100 Alexandroupolis, Greece

**Keywords:** advanced ovarian cancer, cytoreductive surgery, chemotherapy, PARP inhibitors, antibody-drug conjugates, immunotherapy, adoptive cell therapy, oncolytic virus, cytokine therapy, platinum-based chemotherapy

## Abstract

Ovarian cancer is the most lethal gynecologic malignancy, which causes 313,959 new cases and 207,252 deaths worldwide annually. The lack of specific symptoms, together with no effective screening tools, results in 75% of patients receiving their diagnosis at an advanced stage. The combination of cytoreductive surgery with platinum-based chemotherapy plays a pivotal role in the treatment of advanced epithelial ovarian cancer, but patients still experience poor long-term survival because of frequent relapses and chemotherapy resistance. The treatment landscape has evolved because bevacizumab and Poly-ADP Ribose Polymerase inhibitors now serve as frontline and maintenance therapies for homologous recombination-deficient tumors. Treatment decisions for recurrent disease depend on platinum sensitivity assessment, which determines the appropriate therapeutic approach, while targeted agents deliver significant benefits to specific patient groups. The development of antibody-drug conjugates such as mirvetuximab soravtansine and immunotherapy, including checkpoint inhibitors and cancer vaccines, demonstrates promising investigative potential. The precision of therapy improves through the use of emerging biomarkers and molecular profiling techniques. The future management of this disease may change because of innovative approaches that include adoptive cell therapy, cytokine therapy, and oncolytic viruses. The progress made in ovarian cancer treatment still faces challenges when it comes to drug resistance, survival improvement, and life quality preservation. The development of translational research alongside clinical trials remains essential to bridge treatment gaps while creating personalized therapies based on molecular and clinical tumor characteristics.

## 1. Introduction

Ovarian cancer stands as the most lethal gynecologic malignancy, which causes 313,959 new cases and 207,252 deaths worldwide annually [1]. The disease mainly affects women after menopause, while the highest incidence occurs among women between the ages of 55 and 64 years. The majority of ovarian cancer cases are epithelial in origin. The lack of specific symptoms, together with no effective screening tools, results in 75% of patients receiving their diagnosis at stages III or IV, when the five-year survival rate drops to 17–28% [2,3].

The standard treatment protocol for advanced ovarian cancer starts with cytoreductive surgery (CRS) followed by platinum-based systemic chemotherapy, typically combining carboplatin and paclitaxel [4]. The goal of CRS is to achieve complete cytoreduction (CC-0) and surgically remove all macroscopic disease from the peritoneal cavity. In order to achieve maximum results, extensive peritonectomies and visceral resections are performed, which require knowledge of advanced surgical techniques [5,6,7,8,9].

The initial response to treatment reaches 60–80%, but patients experience a 70% chance of relapse during the first three years post-treatment [1]. Targeted therapy treatment with poly-ADP ribose polymerase (PARP) inhibitors demonstrates effectiveness in patients with breast cancer (BRCA) genes mutations or homologous recombination deficiencies [4]. The addition of anti-angiogenic agents like bevacizumab to treatment protocols leads to better progression-free survival, and the approval of antibody-drug conjugates, such as mirvetuximab soravtansine, provides new treatment possibilities for patients with platinum-resistant disease who express folate receptor alpha [1,10].

The recent advancements in ovarian cancer treatment demonstrate why personalized medicine plays a crucial role in managing advanced ovarian cancer, while demanding further research to develop individualized treatment approaches for better patient results. Our thorough review aims to highlight the current adjuvant treatment options for both advanced ovarian cancer and its recurrence and to investigate emerging and future therapeutic directions in the field.

## 2. First-Line Management of Advanced Ovarian Cancer

### 2.1. Chemotherapy

Primary cytoreductive surgery continues to play a pivotal role in the treatment of advanced epithelial ovarian cancer, with the main therapeutic goal being the achievement of optimal debulking [11]. The usual first-line treatment for patients with stage II or higher ovarian cancer with primary surgical cytoreduction is intravenous platinum-based chemotherapy, such as carboplatin or cisplatin, and taxanes like docetaxel or paclitaxel [12,13]. This regimen is usually given for six cycles, with three-week intervals [12,13]. It has been the standard of care for more than the last 20 years and has significantly improved patient survival rates [12,13]. A higher incidence of severe toxicities has been associated with alternative chemotherapy regimens, such as carboplatin every three weeks together with weekly paclitaxel or both drugs weekly [14,15,16]. Specifically, these substitutes have demonstrated grade 3 or 4 toxicities at 63% and 53%, respectively, in contrast to the 42% toxicity rate of the conventional carboplatin plus paclitaxel every three weeks regimen [17].

Neoadjuvant chemotherapy (NACT) is a well-established treatment strategy for patients with newly diagnosed advanced epithelial ovarian cancer (FIGO stages III–IV), when the probability of optimal cytoreduction through primary cytoreductive surgery is low or the perioperative risk is considered high [18,19]. In these cases, the administration of three to four cycles of chemotherapy (usually platinum/taxane) aims to reduce the disease volume and improve the possibility of optimal surgical intervention [20]. Interval cytoreductive surgery is ideally recommended after ≤4 cycles of NACT, as extension beyond this has not been studied prospectively and should be based on individualized, patient-centered factors [19,21,22,23]. After completion of interval cytoreductive surgery, it is recommended to administer adjuvant chemotherapy for a total of 6 cycles, including the cycles received preoperatively, while the exact number of cycles can be modified individually depending on the patient and the treatment response [19]. The decision between primary and interval cytoreductive surgery should be made within a multidisciplinary team, guided by comprehensive imaging, clinical performance status, and the expertise of a gynecologic oncologist [19].

### 2.2. Angiogenesis and Bevacizumab

Angiogenesis plays a critical role in the development and progression of ovarian cancer, representing a crucial biological mechanism targeted by modern therapeutic approaches [24,25]. Bevacizumab is a humanized monoclonal antibody that specifically targets vascular endothelial growth factor (VEGF) by blocking its binding to the VEGFR-1 and VEGFR-2 receptors on endothelial cells [26]. In this way, it inhibits angiogenesis and reduces vascular permeability, helping to inhibit tumor growth and metastasis in advanced ovarian cancer [26].

The efficacy of bevacizumab as part of first-line and maintenance therapy in combination with platinum–taxane chemotherapy for advanced ovarian cancer was thoroughly evaluated in two large, randomized phase III studies: GOG-0218 (NCT00262847) and ICON7 (NCT00483782) [27,28,29,30]. The GOG-0218 trial enrolled 1873 women with newly diagnosed, advanced (incomplete resectable stage III or IV) ovarian, fallopian tube, or peritoneal cancer and investigated the addition of bevacizumab (15 mg/kg) to paclitaxel-carboplatin chemotherapy followed by maintenance therapy for up to 15 months [27,28]. Results showed a significant improvement in median progression-free survival (PFS) of 3.8 months (14.1 vs. 10.3 months), but without a comparable improvement in overall survival (OS) [27,28].

Similarly, the ICON7 study enrolled 1528 women with newly diagnosed ovarian cancer, including both high-risk cases of early-stage disease (FIGO stage I–IIa with grade 3 tumours or clear cell histology) and more advanced stages (FIGO stage IIb–IV), who were treated with bevacizumab at a lower dose (7.5 mg/kg) and for a shorter duration (12 months) [29,30]. The study recorded an improvement in PFS of 1.7 months, and in a pre-specified subgroup of high-risk patients (stage III with >1 cm residual disease or stage IV), a benefit in both PFS (18.1 vs. 14.5 months) and OS (39.3 vs. 34.5 months) was observed [29,30]. The results of the two studies contributed to the approval of bevacizumab by the European Medicines Agency (EMA) in 2011 as part of first-line and maintenance treatment in advanced ovarian cancer, and the US FDA approved its use for this indication in 2018 [31]. Despite the clear improvement in PFS, the absence of a significant benefit on overall survival and associated toxicity (e.g., hypertension, gastrointestinal perforation, proteinuria) has led to variation in its use between countries and the need to individualize the treatment strategy [27,28,29,30].

The multicenter randomized phase III trial AGO-OVAR 17/BOOST (NCT01462890) investigated the optimal duration of bevacizumab in combination with first-line chemotherapy (carboplatin-paclitaxel) in patients with advanced ovarian, fallopian tube, or peritoneal cancer (FIGO stage IIb–IV) [32]. A total of 927 women were randomized to receive bevacizumab for either 15 months or 30 months. Results showed that extending treatment to 30 months provided no additional benefit in either PFS or OS [32] In addition, adverse events (such as hypertension, thromboembolic events, and proteinuria) were slightly increased in the 30-month group. Therefore, the 15-month duration of administration remains the standard therapeutic practice [32].

Table 1 provides an overview of pivotal phase 3 trials assessing the efficacy and safety of bevacizumab in the first-line treatment of ovarian cancer.

### 2.3. Poly-ADP Ribose (PARP) Inhibitors in First-Line Therapy

PARP inhibitors represent a major advancement in the treatment of ovarian cancer, particularly in tumors with impaired homologous recombination (HR) DNA repair [33]. By blocking the repair of single-strand DNA breaks (SSBs), PARP inhibitors lead to the accumulation of unrepaired lesions that convert into double-strand breaks (DSBs), which cannot be effectively repaired in the absence of functional HR, resulting in synthetic lethality and cancer cell death [33,34,35]. This mechanism is especially relevant in tumors harboring BRCA1/2 mutations [33]. Approximately 41–50% of epithelial ovarian carcinomas are estimated to exhibit homologous recombination deficiency (HRD), although prevalence varies depending on the assessment method (germline or somatic mutations, or HRD score) and histologic subtype [36]. High-grade serous ovarian carcinoma (HGSOC) demonstrates the highest HRD prevalence, but notable rates are also observed in endometrioid and clear cell subtypes [36]. Therefore, PARP inhibitors constitute a key targeted therapeutic option for a substantial proportion of patients with ovarian cancer [36].

The introduction of PARP inhibitors as first-line maintenance therapy in ovarian cancer has altered the standard clinical practice and the disease’s evolution [34]. The SOLO1/GOG 3004 (NCT01844986) phase III randomized trial evaluated olaparib as a first-line maintenance therapy in patients with newly diagnosed, advanced (FIGO stage III–IV), high-grade serous or endometrioid ovarian, fallopian tube, or primary peritoneal cancer, who had a BRCA1 and/or BRCA2 mutation and had achieved a complete or partial response to platinum-based chemotherapy [37]. Patients received maintenance therapy with olaparib for up to 2 years (or until disease progression or unacceptable toxicity), with a median treatment duration of 24.6 months [38]. Olaparib reduced the risk of disease progression or death by 70% compared to placebo (HR: 0.30), extending median progression-free survival (PFS) to 56 months versus 13.8 months [39]. At 7-year follow-up, 67% of patients in the olaparib group were alive, compared to 46.5% in the placebo group (OS HR: 0.55), with no new safety concerns reported, supporting its role in achieving long-term remission and potentially cure [38].

Moreover, the PRIMA/ENGOT-OV26/GOG-3012 (NCT02655016) phase III trial evaluated niraparib as first-line maintenance in patients with newly diagnosed advanced ovarian cancer at high risk of recurrence who responded to platinum-based chemotherapy [40]. Patients received niraparib maintenance therapy for up to 36 months [41]. In the primary analysis, among 733 randomized patients, niraparib significantly improved PFS both in the HRD population (21.9 vs. 10.4 months; HR: 0.43) and in the overall population (13.8 vs. 8.2 months; HR: 0.62) [40]. At a median follow-up of 3.5 years, the PFS benefit was sustained and was also observed in HRD-negative (HRp) patients [40]. At 5 years, HRD-positive patients were twice as likely to remain progression-free with niraparib [41]. No difference in OS was observed, and no new safety concerns emerged during long-term follow-up [41].

Following PRIMA, the ATHENA–MONO trial (NCT03522246) studied rucaparib as first-line maintenance in a broader population of patients with newly diagnosed advanced high-grade ovarian cancer, regardless of BRCA or HRD status [42]. In this phase III study, 538 patients were randomized (4:1) to rucaparib or placebo after response to platinum-based chemotherapy [42]. Patients were treated with maintenance rucaparib for a maximum duration of 24 months or until disease progression or unacceptable toxicity [43]. Notably, 35% of patients in the rucaparib arm completed the full 2-year treatment course, compared with 17% in the placebo group [43]. Rucaparib significantly improved progression-free survival both in the HRD-positive group (28.7 vs. 11.3 months; HR: 0.47) and the overall population (20.2 vs. 9.2 months; HR: 0.52), with benefits also observed in HRD-negative patients [42]. The safety profile was manageable, with anemia and neutropenia being the most common grade ≥ 3 adverse events. Overall survival data remain immature, without a demonstrated OS benefit to date [43].

In the context of studies evaluating PARP inhibitors as a first-line treatment, the VELIA/GOG-3005 (NCT02470585) study evaluated veliparib in combination with first-line chemotherapy and as maintenance therapy in patients with newly diagnosed stage III–IV high-grade serous ovarian cancer [44]. Patients received 6 cycles of induction chemotherapy with or without veliparib, followed by maintenance therapy with veliparib for up to 30 additional cycles (approximately 2 years) [44]. Among 1140 participants, veliparib administered throughout treatment significantly improved progression-free survival across all subgroups: 34.7 vs. 22.0 months in BRCA-mutated patients (HR 0.44), 31.9 vs. 20.5 months in the HRD cohort (HR 0.57), and 23.5 vs. 17.3 months in the overall population (HR 0.68) [44]. Common grade ≥ 3 adverse events included anemia, thrombocytopenia, and gastrointestinal toxicity, particularly when veliparib was combined with chemotherapy [44]. The benefit of veliparib during induction only, without maintenance, was less clear [44].

Expanding upon the data from first-line PARP inhibitor therapies, the PAOLA-1 study (NCT02477644) evaluated the combination of olaparib and bevacizumab as maintenance treatment in patients with newly diagnosed advanced ovarian cancer who had achieved a clinical response following first-line platinum-taxane chemotherapy with bevacizumab [45]. Patients received oral olaparib (300 mg twice daily) for up to 24 months and intravenous bevacizumab (15 mg/kg every 3 weeks) for a total of 15 months [45]. The combination significantly improved PFS compared to bevacizumab alone (22.1 vs. 16.6 months, HR 0.59), with a pronounced benefit in HRD-positive tumors (37.2 vs. 17.7 months in BRCA-mutated; 28.1 vs. 16.6 months in BRCA wild-type/HRD+) [45]. The final OS analysis confirmed a clinically significant benefit in the HRD-positive population (5-year OS: 65.5% vs. 48.4%, HR 0.62), establishing the combination as a standard of care for this subgroup [46]. Collectively, these findings have established PARP inhibitors as a cornerstone of maintenance therapy in advanced ovarian cancer, particularly among patients with homologous recombination deficiency.

Table 2 summarizes the key features and findings of major phase III trials evaluating PARP inhibitors as maintenance therapy in first-line advanced ovarian cancer.

## 3. Management of Recurrent Disease

Despite achieving a complete response in about 80% of ovarian cancer patients after initial treatment, the majority of patients relapse within about 18 months, with chemoresistance being a major cause [47]. Specifically, more than 60% of patients with residual disease < 1 cm (optimal debulking) and approximately 80% of those with residual disease > 1 cm (suboptimal debulking) experience recurrence within this time [47]. During the relapse phase, the treatment strategy is mainly based on the sensitivity of the disease to platinum, with second-line chemotherapy chosen accordingly [47,48,49]. Classification of relapse based on Progression-Free Interval (PFI) includes platinum-resistant disease (relapse within 6 months of treatment), platinum-sensitive disease (relapse > 6 months), and further subcategorization into partially sensitive (6–12 months) and highly sensitive (>12 months), with relapse during first-line treatment classified as platinum-refractory [47,48,49].

### 3.1. Platinum-Sensitive Recurrent Ovarian Cancer

The choice of second-line chemotherapy depends on the tumor’s sensitivity to platinum-based agents [48,49,50]. Specifically, the selection of second-line chemotherapy for recurrent platinum-sensitive ovarian cancer is guided by tumor platinum sensitivity and patient-specific contraindications [48,49,50]. Patients with a high likelihood of response and without significant platinum hypersensitivity typically receive combination regimens involving carboplatin paired with paclitaxel, gemcitabine, or pegylated liposomal doxorubicin (PLD), which have demonstrated superiority over monotherapy in terms of PFS and OS [48,49,50]. Despite their clinical benefit, combination therapies may cause increased toxicity; therefore, regimen choice should carefully consider patient performance status and prior treatment-related toxicities [48,50]. In case of severe hypersensitivity reactions to platinum, desensitization protocols or alternative regimens, such as the combination of PLD with trabectedin, can be implemented, although these have a lower survival benefit compared to platinum-based regimens [48,50]. In addition, patients who respond to platinum-based regimens can then receive maintenance therapy with PARP inhibitors or bevacizumab to further improve outcomes [48,49,50].

The OCEANS study (NCT00434642), a randomized, double-blind, placebo-controlled phase III trial, evaluated the addition of bevacizumab to carboplatin and gemcitabine in patients with platinum-sensitive recurrent ovarian, primary peritoneal, or fallopian tube cancer [51,52]. Gemcitabine and bevacizumab, followed by bevacizumab maintenance until disease progression, significantly improved progression-free survival (PFS: 12.4 vs. 8.4 months, HR = 0.484) and response rate (78.5% vs. 57.4%) compared to Gemcitabine + placebo, with no new safety issues, although increased rates of grade ≥ 3 hypertension and proteinuria were observed in the bevacizumab group [51]. The final analysis of OS in the OCEANS trial showed no statistically significant difference between the groups (OS: 33.6 vs. 32.9 months, HR = 0.95, *p* = 0.65), while safety remained consistent with previous data [52]. In addition, the GOG-0213 study (NCT00565851) confirmed the benefit of adding bevacizumab to standard chemotherapy with carboplatin and paclitaxel in patients with platinum-sensitive relapse, demonstrating an improvement in median OS (42.2 vs. 37.3 months, HR = 0.823) according to sensitive analysis based on corrected treatment-free interval value. At the same time, increased grade ≥ 3 toxicity was reported in the bevacizumab group, in particular hypertension and proteinuria [53].

Beyond antiangiogenic strategies, significant advances have been made in targeting DNA repair pathways in ovarian cancer in this setting. The SOLO2/ENGOT-Ov21 study (NCT01874353) showed that maintenance treatment with olaparib (300 mg twice daily) in patients with platinum-sensitive, recurrent ovarian cancer and BRCA1/2 mutation significantly improved progression-free survival (19.1 vs. 5.5 months, HR = 0.30, *p* < 0.0001) compared to placebo. The most common grade ≥ 3 adverse events were anemia, fatigue, and neutropenia [54]. Final analysis showed a trend in favor of overall survival in favor of olaparib (51.7 vs. 38.8 months, HR = 0.74), but without statistical significance, probably due to the extended administration of PARP inhibitors in the placebo group after disease progression [55].

Furthermore, the ENGOT-OV16/NOVA study (NCT01847274) showed that niraparib as maintenance therapy in patients with platinum-sensitive, recurrent ovarian cancer significantly prolongs PFS in both patients with BRCA mutation (21.0 vs. 5.5 months, HR = 0.27) and those with HRD-positive tumors without BRCA mutation (12.9 vs. 3.8 months, HR = 0.38) [56]. In the final OS analysis, with a median follow-up >75 months, no statistically significant difference was found, although there was a numerical benefit in the BRCA group (40.9 vs. 38.1 months, HR = 0.85) [57].

Moreover, the ARIEL3 trial (NCT01968213) was a randomized, double-blind, placebo-controlled phase 3 study that evaluated maintenance treatment with rucaparib in patients with platinum-sensitive recurrent ovarian cancer. [58,59] Administration of rucaparib significantly improved PFS in all analysis groups: in patients with BRCA mutation (16.6 vs. 5.4 months, HR = 0.23, *p* < 0.0001), in patients with HRD (13.6 vs. 5.4 months, HR = 0.32, *p* < 0.0001), and in the whole population (10.8 vs. 5.4 months, HR = 0.36, *p* < 0.0001) [58]. The main grade ≥3 adverse events were anemia and liver enzyme elevations [58]. In the final analysis, after a median follow-up of 77 months, rucaparib showed a trend in favor of OS, although the outcome was affected by the use of PARP inhibitors in patients in the placebo group [59].

Furthermore, secondary cytoreduction in patients with platinum-sensitive recurrent epithelial ovarian cancer is a subject of intensive investigation in the last years, with conflicting results regarding the benefit to survival [60,61,62,63]. The GOG-0213 study (NCT00565851) showed that the addition of secondary cytoreduction before chemotherapy did not improve OS compared to chemotherapy alone (median OS: 50.6 vs. 64.7 months, HR = 1.29, *p* = 0.08), despite achieving complete resection in 67% of patients [60].

In contrast, the DESKTOP III trial (NCT01166737) demonstrated a significant benefit to overall survival in favor of the surgery group (median OS: 53.7 vs. 46.0 months, HR = 0.75, *p* = 0.02), with the greatest benefit seen in patients with complete resection (median OS: 61.9 months) [61]. Similarly, the Chinese SOC-1 study (NCT01611766) demonstrated significant prolongation of progression-free survival (PFS: 17.4 vs. 11.9 months, HR = 0.58, *p* < 0.0001), while the final analysis of overall survival showed a trend in favor of the surgery after crossover adjustment (adjusted HR = 0.76) [62,63]. In all studies, achieving complete cytoreduction emerged as a key prognostic factor [61,62,63]. Therefore, the selection of patients with a probability of complete resection (based on AGO, iMODEL, or PET-CT criteria) is critical, and surgery should be performed in specialized centers with experience [61,62,63]. Secondary cytoreduction is indicated for selected patients with recurrent, platinum-sensitive disease, provided complete resection is possible and of appropriate functional status [61,62,63].

### 3.2. Platinum-Resistant Recurrent Ovarian Cancer

The management of recurrent platinum-resistant ovarian cancer remains a clinical challenge, as the prognosis is poor and response rates to available therapies are low [48,49]. In this setting, monotherapy with non-platinum chemotherapeutic agents, such as weekly paclitaxel, topotecan, PLD, or gemcitabine, is the main therapeutic option, with objective response rates in the range of only 8–20% [47,48,49,50]. Although drug combinations show slightly higher response and PFS rates, they are associated with increased toxicity with no clear benefit in OS, leading to a preference for monotherapy [48].

The addition of bevacizumab to chemotherapy monotherapy has demonstrated clinical benefit in this group of patients [64]. In the AURELIA trial, the combination of bevacizumab with chemotherapy (PLD, paclitaxel, or topotecan) significantly improved PFS (6.7 vs. 3.4 months, HR = 0.48, *p* < 0.001) and objective response rate (27.3% vs. 11.8%, *p* = 0.001) compared to chemotherapy alone [64]. Although no statistically significant improvement in OS was recorded (16.6 vs. 13.3 months, HR = 0.85, *p* = 0.174), the overall toxicity was acceptable, with severe hypertension and proteinuria being the most common adverse events [64]. The combination of trabectedin with PLD has been approved for the treatment of patients with ovarian cancer who relapse longer than 6 months after their last platinum-based therapy [50]. Personalization of treatment based on general condition, toxicity history, and patient preferences is essential, and in suitable patients, priority should be given to participation in clinical trials [50].

Folate receptor α (FRα) is overexpressed in approximately 80% of cases of high-grade epithelial ovarian cancer, whereas its expression in normal tissues remains limited [65]. Targeted therapy of FRα has emerged as an attractive therapeutic strategy for selected groups of patients [65]. Mirvetuximab soravtansine is an antibody-drug complex (ADC) that combines a monoclonal antibody against FRα with a DM4 cytotoxic load (soravtansine), a microtubule inhibitor [66]. By selectively targeting cancer cells that overexpress FRα, it allows intracellular release of the cytotoxic agent, reducing the effect on normal tissues [65].

The SORAYA study (NCT04296890), a single-arm phase III trial, evaluated mirvetuximab in patients with platinum-resistant ovarian cancer and high FRα expression (≥75% of strongly expressed, 2+/3+ cancer cells) [66]. An objective response rate (ORR) of 32.4% and a median duration of response (DOR) of 6.9 months were observed [66]. The safety profile was favorable, with the most common adverse events including mild to moderate keratopathy, blurred vision and fatigue, with a relatively low incidence of serious adverse events [66]. The randomized MIRASOL phase III trial (NCT04209855) confirmed these findings. They enrolled patients with platinum-resistant disease and high FRα expression (≥75% of cells with ≥2+ intensity) [67]. Mirvetuximab achieved a significantly higher ORR versus the chemotherapy of choice (42% vs. 16%) and improved median PFS (5.6 vs. 4 months, HR = 0.65) [67]. A trend towards improved overall survival was also recorded (HR = 0.67) [67]. Regarding safety, mirvetuximab was consistent with previous reports, showing lower rates of serious adverse events and treatment discontinuations due to toxicity compared to the investigator’s choice of chemotherapy [67]. The most reported adverse events were blurred vision, keratopathy, fatigue and diarrhea, which were generally manageable [67]. Thus, mirvetuximab soravtansine is an effective and well-tolerated therapeutic option for patients with platinum-resistant ovarian cancer and high FRα expression, enhancing the therapeutic arsenal in this challenging clinical setting.

In addition to targeting FRα, the emerging strategy of targeting HER2 is of great research interest. As part of the non-randomized, multicenter, phase II DESTINY-PanTumor02 study (NCT04482309), trastuzumab deruxtecan (T-DXd) was evaluated in 40 patients with platinum-resistant, HER2-expressing ovarian cancer (IHC 3+ or 2+), demonstrating encouraging results with an overall response rate (ORR) of 45% and a median duration of response (DOR) of 11.3 months [68]. In patients with high HER2 expression (IHC 3+), the ORR reached 63.6% with a median DOR of 22.1 months [68]. The safety profile was considered manageable, with the most common adverse events being nausea, fatigue, and hematological toxicities [68]. These findings support the potential of T-DXd in biologically selected subgroups of patients with resistant disease.

## 4. Emerging and Future Therapeutic Directions

### 4.1. Immunotherapy Approaches

#### 4.1.1. Immune Checkpoint Inhibitors

Despite significant advances in the therapeutic management of ovarian cancer, the efficacy of immunotherapeutic interventions with immune checkpoint inhibitors remains under intense investigation. Pembrolizumab, an anti-PD1 agent, received FDA approval in 2020 for the treatment of patients with unresectable or metastatic solid mutational burden-high tumors (TMB-H, ≥10 mutations/Mb) who develop disease progression after previous treatment and for whom no satisfactory alternative treatment options are available, regardless of histological type, based on data from the Phase II KEYNOTE-158 study and other relevant analyses, highlighting the importance of molecular tumor characteristics and personalization of the immunotherapeutic strategy [69].

Several randomized phase III studies have evaluated the administration of immune checkpoint inhibitors at various phases of the disease, aiming to improve clinical outcomes while at the same time providing critical information on the biology of the disease and further development of targeted immunotherapeutic interventions [70,71,72,73,74,75,76,77,78,79,80,81,82]. Table 3 summarizes the key features and findings of major phase 3 clinical trials involving immune checkpoint inhibitors in ovarian cancer.

#### 4.1.2. Cancer Vaccines

**Figure 1:**
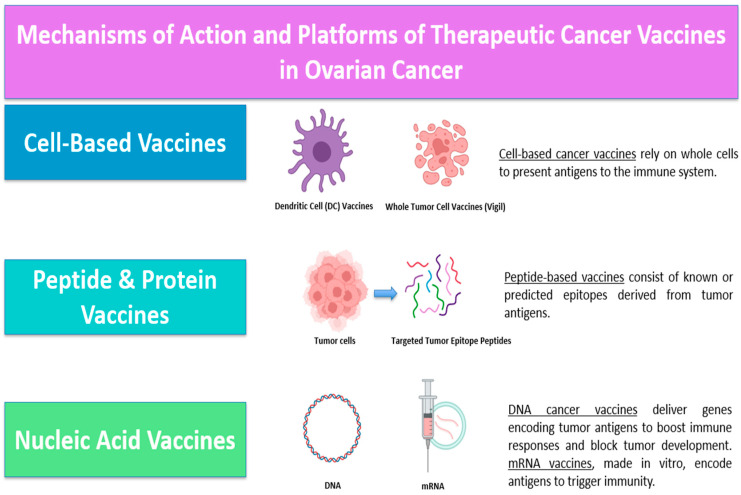
Mechanisms of action and platforms of therapeutic cancer vaccines in ovarian cancer.

The use of immunotherapeutic cancer vaccines represents a promising area, aiming to activate the adaptive immune system against neoantigens or tumor-associated antigens, thereby achieving immune memory and eliminating disease with minimal off-target toxicity [83]. The main vaccine platforms include cell-based vaccines, peptide-based vaccines, and nucleic acid (DNA or mRNA) vaccines (Figure 1) [84]. Following advances in mRNA technology during the COVID-19 pandemic, there has been increased interest in the development of personalized vaccines [83].

Furthermore, in ovarian cancer, particular emphasis is given to dendritic cell (DC) vaccines, which activate T cells through antigen presentation [85,86]. Phase II clinical trials have shown positive results for their safety and immunogenicity [86]. In addition, the TPIV200 vaccine targeting the FRα, in combination with PD-L1 antibodies, has been shown to enhance the immune response and can overcome resistance mechanisms [86]. At the same time, the Vigil vaccine, based on autologous cancer cells expressing GM-CSF and inhibiting furin, has shown encouraging results in recurrent ovarian cancer patients with tumors expressing PD-L1 in a phase I/II study, giving a benefit in PFS in combination with durvalumab [84,85,87].

Moreover, new platforms such as the survivin-targeted vaccine OVM-200 and Maveropepimut-S (MVP-S), which activate specific anti-cancer T-cell responses independent of the presence of platinum sensitivity, are also being investigated in clinical trials [83]. Recent phase II clinical trials, such as TEDOVA (NCT04713514) and DOVACC (NCT04742075), are investigating the role of neoantigenic or telomerase-targeted therapeutic vaccines (OSE2101, UV1) in combination with PD-1/PD-L1 inhibitors and PARP inhibitors in patients with recurrent ovarian cancer, aiming to enhance immune memory and prolong disease-free survival [88,89]. Preliminary results are expected with great interest [88,89]. Despite the relative safety of the vaccines, their overall clinical efficacy remains limited, with one meta-analysis reporting an ORR of only 4% in patients with advanced or recurrent ovarian cancer [85]. Major challenges include the heterogeneity of the tumor microenvironment, the difficulty in preparing autologous DCs, and the high cost of production [85]. Overall, therapeutic vaccinations represent a promising but still developing strategy, with the need for further research to personalize and enhance clinical efficacy [85].

#### 4.1.3. Adoptive Cell Therapy (ACT)

ACT is also emerging as a promising immunotherapeutic strategy in ovarian cancer, aiming to enhance the anti-cancer immune response through the ex vivo activation and genetic modification of T lymphocytes [83,90]. ACT involves the isolation and culture of specialized T cells, such as tumor-infiltrating lymphocytes (TILs) or peripheral T cells, and their reinfusion into the patient for the direct destruction of cancer cells [85]. In recurrent ovarian cancer, administration of TILs has been associated with disease stabilization and increased clinical response rates [83,91,92]. In parallel, significant advances have been made in the genetic engineering of T cells, through technologies such as chimeric antigen receptor (CAR-T) and T-cell receptor (TCR-T) therapies [84]. CAR-T cells target surface tumor antigens via antibody fragments, while TCR-T cells recognize peptide antigens presented by HLA molecules, enabling the targeting of tumors even with low surface antigen expression [84].

Nevertheless, despite the progress, the clinical implementation of ACT faces significant challenges, including the immunosuppressive tumor microenvironment, the technical complexity of cell production, high costs, and the management of potential severe adverse events [83]. Further well-designed clinical trials are therefore essential to precisely define the efficacy and safety profile of ACT in ovarian cancer [85].

#### 4.1.4. Oncolytic Virus

Another immunotherapeutic intervention under investigation is oncolytic viruses, which can selectively infect cancer cells, induce immediate cell lysis, and release antigens that stimulate a broader anti-cancer immune response [83]. One characteristic agent is talimogene laherparepvec (T-VEC), a genetically modified herpes virus, which has been evaluated for the treatment of unresectable or advanced melanoma, achieving high rates of overall response through intra-tumoral injection [83,84,85,93,94]. In ovarian cancer, the use of oncolytic viruses is being evaluated both as monotherapy and in combination with other immunotherapeutic agents [84,94]. Of great interest is the use of modified adenoviruses (such as CRAd), as well as strategies to combine tumor-catalytic viruses with cell therapies or radiotherapy, enhancing tumor immunogenicity and promoting the efficacy of the antitumor response [84,94].

#### 4.1.5. Cytokine Therapy

Another promising yet challenging area of immunotherapy in ovarian cancer is cytokine therapy, which aims to enhance the immune response by administering or modifying specific cytokines, such as IL-2, IL-12 and IL-15, that activate natural killer (NK) and cytotoxic T lymphocytes [84]. IL-2 was the first molecule to be clinically evaluated to enhance T-cell activity; however, its toxicity and non-targeted action limited its use [84]. Newer approaches, such as the IL-15-based molecule N-803, have shown greater biological activity [95]. At the same time, IL-12 and IL-18 emerged as potent enhancers of inflammatory and anticancer responses in preclinical models [84,96]. In addition to the standard cytokines, other molecules, such as IL-4, IL-9, and IL-24, are of research interest; they seem to act at different levels of the anti-cancer immune axis [97,98,99]. Despite promising data from preliminary studies, the widespread clinical application of cytokine therapy remains a challenge, mainly due to the pleiotropic nature of their effects and the need for better targeting [84].

### 4.2. Antibody-Drug Conjugates (ADCs)

ADCs have emerged as a promising therapeutic approach in ovarian cancer, combining targeted recognition of surface antigens of cancer cells with selective delivery of cytotoxic payloads [100]. The expression of specific antigens, such as FRα, MUC16/CA125, and TROP2 in ovarian cancer cells has led to the development of novel ADCs with improved efficacy and safety profiles [101].

#### 4.2.1. Targeting Folate Receptor Alpha

Antibody-directed drugs targeting FRα are a highly promising strategy in ovarian cancer, with many molecules in different stages of clinical development. Mirvetuximab soravtansine was the first ADC to show significant clinical activity in platinum-resistant ovarian cancer. In the phase Ib/2 FORWARD II study (NCT02606305), it was evaluated in combination with bevacizumab, carboplatin PLD, or pembrolizumab, demonstrating safe combination therapy with no new toxicity [102]. In particular, the combination with bevacizumab in FRα-positive patients achieved an ORR of 44% and a median PFS of 8.2 months, with a good safety profile [103].

In parallel, farletuzumab, an anti-FRα monoclonal antibody, was investigated in two studies in platinum-sensitive recurrent ovarian cancer [104,105]. In the phase III study (NCT00318370), farletuzumab in combination with carboplatin and taxane (paclitaxel or docetaxel) did not significantly improve progression-free survival (PFS) compared with placebo, although subgroups of patients with low CA-125 levels showed a benefit [104]. In a subsequent phase II study (NCT02289950), farletuzumab in combination with carboplatin and paclitaxel or PLD also did not significantly improve PFS compared with placebo in patients with low CA-125 levels [105].

Moreover, a new ADC, luveltamab tazevibulin (luvelta), also targets FRα with a stable cleavable linker and a 3-aminophenyl hemiasterlin warhead [106]. In the phase I STRO-002-GM1 study (NCT03748186), luvelta showed remarkable antitumor activity in recurrent ovarian cancer with FRα expression > 25%, with manageable toxicity (neutropenia, arthralgia, anemia) [106]. In addition, in the phase I STRO-002-GM2 study (NCT05200364) it is being tested in combination with bevacizumab in patients with recurrent high-grade epithelial ovarian cancer, while in the phase II/III REFRaME-01 study (NCT05870748) it is being compared with the treatment of choice in patients with recurrent platinum-resistant disease expressing FRα [107,108].

Finally, Rinatabart sesutecan (Rina-S, PRO1184), an ADC loaded with the topoisomerase 1 inhibitor exatecan, is currently under evaluation in ovarian cancer with varying levels of FRα expression [109]. In the phase I/II RAINFOL-01 study (NCT05579366), Rina-S showed promising results in patients with recurrent ovarian cancer, regardless of FRα expression levels [110]. In addition, the phase III RAINFOL-02 study (NCT06619236) is ongoing, evaluating Rina-S in patients with platinum-resistant ovarian cancer compared to the investigator’s choice of treatment, while in 2024, Rina-S received a Fast Track designation from the FDA for FRα-positive platinum-resistant high-grade serous or endometrioid ovarian cancer [111].

#### 4.2.2. Targeting Trophoblast Cell Surface Antigen 2 (TROP2)

Another type of ADC targets the TROP2 antigen. Datopotamab deruxtecan (Dato-DXd), an anti-TROP2 ADC, is being evaluated in several studies in advanced solid tumors, including ovarian cancer [112].

The phase II TROPION-PanTumor03 study (NCT05489211) evaluates the efficacy of Dato-DXd as a monotherapy and in combination with other agents in various tumors, including ovarian cancer [112]. Recent ESMO 2024 data indicate that Dato-DXd monotherapy produced an objective response rate (ORR) of 42.9% and a median progression-free survival (PFS) of 5.8 months in patients with recurrent unresectable advanced/metastatic high-grade carcinoma of the ovaries, fallopian tubes, or primary peritoneal carcinoma, whose disease had progressed after ≥1 line of platinum-based chemotherapy, and the toxicity profile was manageable [112].

In parallel, sacituzumab tirumotecan (sac-TMT), an ADC carrying a belotecan-derivative topoisomerase I inhibitor, showed promising efficacy in patients with heavily pre-treated advanced ovarian and endometrial cancer in the phase II KL264-01 study (NCT04152499), with objective response rates (ORRs) of 40% and 34.1%, respectively [113]. In addition, sacituzumab govitecan, an approved anti-TROP2 ADC in other indications, is currently under evaluation in a phase II study (NCT06028932) in recurrent or persistent platinum-resistant epithelial ovarian, fallopian tube, or primary peritoneal carcinoma. Finally, SHR-A1921, a novel ADC that binds an anti-TROP2 monoclonal antibody to a topoisomerase I inhibitor via a tetrapeptide linker, demonstrated encouraging anti-cancer activity and manageable safety in heavily pretreated patients with platinum-resistant ovarian cancer in a phase I study (NCT05154604) [114].

#### 4.2.3. Targeting Cadherin 6 (CDH6)

Cadherin 6 (CDH6), a cell adhesion protein, is overexpressed in most epithelial ovarian carcinomas and is associated with a poor prognosis. Raludotatug deruxtecan (R-DXd, DS-6000a) is an anti-CDH6 antibody-drug conjugate (ADC) that selectively targets CDH6-overexpressing cells, causing intracellular release of a topoisomerase I inhibitor and cell apoptosis [115,116]. In a phase I study (NCT04707248), R-DXd showed an acceptable safety profile and early efficacy signals in patients with advanced ovarian cancer, the majority (88.9%) of whom had platinum-resistant disease [115,116]. Based on these promising data, R-DXd is being further evaluated in the phase II/II REJOICE-Ovarian01 study (NCT06161025), which is comparing R-DXd to the investigator’s choice of chemotherapy (gemcitabine, paclitaxel, topotecan, or pegylated liposomal doxorubicin) in patients with platinum-resistant high-grade ovarian, peritoneal, or fallopian tube cancer [117].

#### 4.2.4. Targeting Claudin 6 (CLDN6)

TORL-1-23 is a novel antibody-drug conjugate (ADC) that targets the tumor-associated protein Claudin 6 (CLDN6), which is overexpressed in several types of cancer, including ovarian, endometrial, and testicular cancer [118].

Researchers evaluated TORL-1-23 in a phase I study (NCT05103683) in patients with advanced ovarian, endometrial, testicular, and lung cancer [118]. TORL-1-23 demonstrated a manageable safety profile, with fatigue, neuropathy, and alopecia as the most common adverse events [118]. In patients with CLDN6-positive ovarian cancer, the response rate (ORR) reached 67% at the dose of 2.4 mg/kg and 50% at 3.0 mg/kg. The study continues in patients with CLDN6+ ovarian cancer and non-small cell lung cancer (NSCLC) [118].

#### 4.2.5. Targeting Mesothelin (MSLN)

Mesothelin (MSLN) is a target with increased expression in ovarian cancer [119,120]. Anetumab ravtansine, an anti-MSLN ADC, showed an ORR of 27.7% and a median PFS of 5 months in combination with pegylated liposomal doxorubicin in a phase Ib study (NCT02751918) in patients with platinum-resistant epithelial ovarian cancer [119]. Furthermore, in a phase II study (NCT03587311), the combination of anetumab ravtansine with bevacizumab was not superior to paclitaxel/bevacizumab (ORR 18% vs. 55%, respectively), leading to early study termination [120].

#### 4.2.6. Targeting MUC16

MUC16 constitutes a target for the development of ADCs in ovarian cancer because of its overexpression in most epithelial tumors [121]. A phase I study (NCT01335958) evaluated DMUC5754A, an ADC using the microtubule-disrupting agent monomethyl auristatin E (MMAE) as a payload, in patients with advanced platinum-resistant ovarian cancer or unresectable pancreatic cancer [122]. DMUC5754A showed an acceptable safety profile, with main adverse events being fatigue, peripheral neuropathy, and nausea, and demonstrated early anticancer activity, especially in patients with high MUC16 expression [122]. In addition, researchers evaluated DMUC4064A, a newer generation anti-MUC16 ADC, in a phase I study (NCT02146313) of patients with platinum-resistant ovarian cancer [121]. DMUC4064A showed good tolerability, with the main toxicity being fatigue and ocular complications. The study recorded clinical benefits (CR, PR, or stable disease ≥6 months) in 42% of patients, particularly those with high MUC16 expression [121]. These data support the ongoing investigation of targeting MUC16 in ovarian cancer using ADCs.

#### 4.2.7. Targeting NaPi2b

NaPi2b (sodium-dependent phosphate transporter) is expressed in a variety of solid tumors, including high-grade ovarian cancer. The phase 1b/2 UPLIFT (NCT03319628) study evaluated Upifitamab rilsodotin (UpRi, XMT-1536), an ADC targeting NaPi2b, in patients with platinum-resistant ovarian cancer [123]. In this study, UpRi demonstrated an overall objective response rate (ORR) of 15.6% in patients with NaPi2b-positive disease (primary endpoint) and 13.1% in the overall population [123]. However, the study failed to meet its pre-specified primary endpoint target, leading to the discontinuation of drug development and suggesting that NaPi2b expression was not a sufficient biomarker for an enriched response to UpRi treatment [123].

#### 4.2.8. Targeting Tissue Factor (TF)

Many solid tumors, including ovarian cancer, express tissue factor (TF) at high levels [124]. Tisotumab vedotin (TV) is an anti-TF antibody-drug conjugate that releases the microtubule-disrupting agent MMAE after the internalization of the complex into cancer cells [124]. The innovaTV 208 (NCT03657043), a phase II, multicenter, open-label study, evaluated the efficacy and safety of TV in patients with platinum-resistant ovarian cancer (PROC) [124]. Patients received TV either every 3 weeks or in a weekly regimen (days 1, 8 and 15 of each 28-day cycle) [124]. In Part B of the study, patients with PROC and ECOG 0-1 received TV intravenously at a dose of 0.9 mg/kg weekly [124]. The primary endpoint for phase B was ORR according to RECIST v1.1 [124]. The study has been completed, and results are expected.

#### 4.2.9. Targeting B7-H4

B7-H4 is a molecule with low expression in normal tissues but high expression in many solid tumors, including ovarian cancer; it is associated with poor prognosis [125,126,127]. AZD8205 is an ADC that combines an anti-B7-H4 antibody with a topoisomerase I (TOP1i) inhibitor [128]. In a Phase I/IIa study (NCT05123482), AZD8205 showed manageable toxicity and early antitumor activity in patients with heavily pre-treated ovarian, breast, and endometrial cancer (ORR 20.9% at doses ≥ 1.6 mg/kg) [125]. SGN-B7H4V is also an ADC with an MMAE load that, in a phase I study (NCT05194072), demonstrated a manageable safety profile and antitumor activity in patients with various tumors, including ovarian cancer [126,129]. Finally, XMT-1660, a novel B7-H4 ADC with a microtubule inhibitor payload, is under investigation in a phase Ib trial (NCT05377996) in patients with breast, endometrial, and ovarian cancer [127].

#### 4.2.10. Targeting HER-2

Disitamab vedotin (RC48) is a newer HER2-directed antibody-drug conjugate (ADC), which combines the humanized disitamab monoclonal antibody with the cytotoxic MMAE payload via a cleavable linker [130]. Early clinical data highlight the significant efficacy of RC48 in gynecological tumors, with response rates of up to 83% in cervical cancer and encouraging levels of response in ovarian and endometrial cancer [130]. It also maintains a favorable safety profile, with mild to moderate adverse events and rare occurrence of serious toxicities [130]. A phase II basket trial (NCT06003231) is focusing on patients with previously treated advanced solid tumors that express HER2 (≥1 IHC), including patients with ovarian and endometrial cancer, in order to evaluate the efficacy of RC48 as a new treatment option in this subgroup of patients with limited available therapies [131].

### 4.3. The Role of the Glucocorticoid Receptor

The glucocorticoid receptor (GR) is a cortisol-regulated transcription factor involved in critical physiological processes such as stress response, metabolic regulation, and immune response [132,133]. In ovarian cancer, GR activation is associated with anti-apoptotic effects, which may reduce the efficacy of chemotherapy and contribute to the development of resistance [132,133]. Relacorilant is a selective modifier of GR that aims to inhibit the undesirable effects of receptor activation without suppressing its broader physiological function [134,135]. In preclinical models, relacorilant appears to restore the apoptotic activity of taxanes and enhance their anticancer efficacy [134,135].

In a phase II study (NCT03776812), the intermittent combination of relacorilant with nab-paclitaxel significantly improved progression-free survival (PFS) and duration of response (DOR) compared with nab-paclitaxel monotherapy in women with recurrent, platinum-resistant epithelial ovarian cancer, with a numerically favorable safety profile [135]. Based on these findings, the phase III ROSELLA study (NCT05257408), a randomized, international, open-label, two-arm study to compare the combination of intermittent relacorilant and nab-paclitaxel versus nab-paclitaxel monotherapy, is now underway [133]. The study includes patients with recurrent, platinum-resistant, high-grade serous or endometrioid epithelial ovarian, primary peritoneal, or fallopian tube carcinoma [133]. The primary endpoint is PFS with an independent panel assessment, while secondary endpoints include OS, objective response rate, and safety [133]. ROSELLA is expected to provide critical data for the final evaluation of relacorilant as a novel strategy to modify glucocorticoid signaling in platinum-resistant ovarian cancer.

## 5. Conclusions

The treatment of advanced epithelial ovarian cancer is multifactorial and demanding, despite significant advances in recent decades. Primary cytoreductive surgery, combined with platinum-based chemotherapy, remains the cornerstone of treatment [11,12,13]. Achieving complete cytoreduction is still a key predictor of overall survival, while neoadjuvant chemotherapy offers a solution for patients with a low chance of primary complete resection or high surgical risk [18,19]. Multidisciplinary assessment and individualized treatment approach are fundamental to optimizing outcomes.

Complementary to surgical and chemotherapeutic interventions, the introduction of antiangiogenic agents, such as bevacizumab, has enhanced therapeutic possibilities. Antiangiogenic therapy with bevacizumab is now part of first line and maintenance treatment, helping to improve progression-free survival, especially in high-risk groups [27,28,29,30]. The absence of a statistically significant benefit in overall survival and potential toxicities necessitates the individualization of its use [27,28,29,30]. A 15-month duration of administration has been established as the clinical standard [32].

Moreover, in the context of maintaining therapeutic response, PARP inhibitors have also gained momentum, reshaping the maintenance strategy [34]. Studies such as SOLO1, PRIMA, ATHENA, VELIA, and PAOLA-1 document their significant contribution to delaying recurrence, even in cases without BRCA mutations [37,38,39,40,41,42,43,44,45,46].

Still, the treatment of recurrence remains a challenge, because it requires individualized approaches that consider platinum sensitivity levels [47,48,49]. In platinum-sensitive recurrence, the re-administration of platinum in combination with other chemotherapeutic drugs and the use of maintenance agents such as PARP inhibitors or bevacizumab has shown benefit. Patient selection for secondary cytoreductive surgery should be based on strict criteria, with the goal of achieving complete resection [48,49,50,51,52,53,54,55,56,57,58,59]. In contrast, in platinum-resistant disease, treatment options are limited, with low response rates and modest survival prolongation [48,49]. In this context, agents such as mirvetuximab soravtansine for patients with high FRα expression and trastuzumab deruxtecan in HER2-positive tumors provide a new perspective [66,67,68].

The need for innovative and more personalized therapeutic strategies has led to intensive investigations of emerging targeted and immunotherapeutic interventions. Immunotherapy, although it has not yet led to spectacular clinical benefits in ovarian cancer, remains an area of intensive research. Phase III studies with immune checkpoint inhibitors (anti-PD-1/PD-L1) such as IMagyn050, JAVELIN, DUO-O, and KEYLYNK-001 have reported conflicting or negative results, highlighting the need for more selective immune predictive biomarkers [70,71,72,77,80,81].

At the same time, vaccine therapies, such as Vigil and neoantigen mRNA-based vaccines, attempt to activate the patient’s immune memory, with early but encouraging results [84,85,86,87]. In addition, adoptive cell therapies such as CAR-T and TCR-T enable precise targeting and destruction of cancer cells, with the main challenges being the technical difficulties and the highly immunosuppressive microenvironment of tumors [83,84].

Beyond checkpoint inhibitors and cell therapies, additional immunotherapeutic approaches are being investigated with the aim of enhancing anticancer immunity through different mechanisms of action. Oncolytic viruses, such as T-VEC and modified adenoviruses, are emerging as innovative immunotherapeutic approaches, enhancing tumor immunogenicity and promoting a multiparametric anticancer response [83,84,85,93,94]. At the same time, cytokine therapy, especially through novel molecules such as IL-15 (N-803), shows encouraging preliminary data, although the complexity of immunological effects requires targeted design for clinical application [84,95].

Furthermore, a rapidly growing field is that of Antibody–Drug Conjugates (ADCs), which combine targeted molecular recognition of tumor surface antigens with the targeted delivery of cytotoxic agents [100]. In addition to mirvetuximab soravtansine, luveltamab tazevibulin, which also targets the folate receptor (FRα), has demonstrated significant efficacy in patients with platinum-resistant disease, particularly those with high expression of the target [106,107,108]. Similar results have been reported with molecules targeting TROP2, such as datopotamab deruxtecan, and Cadherin 6 (CDH6), with raludotatug deruxtecan [112,115,116,117]. Further promising data are emerging for antigenic targets such as CLDN6, MSLN, MUC16/CA125, NaPi2b, TF, B7-H4, and HER2, with ADCs showing high response rates in preliminary clinical trials [118,119,120,121,122,123,124,125,126,127,128,129,130,131].

Another promising approach is the modification of glucocorticoid receptor (GR) signaling, which appears to be related to mechanisms of resistance to chemotherapy [132,133]. The agent relacorilant, a selective GR modulator, enhances the action of taxanes and has shown clinical benefit in patients with platinum-resistant disease, leading to the ongoing Phase III ROSELLA study [133,134,135].

Despite significant advances, the current literature presents substantial limitations. Most available data are derived from small-scale, non-randomized studies with short follow-up durations and heterogeneous inclusion criteria. The lack of large, multicenter randomized trials and long-term survival outcomes limits the ability to draw definitive conclusions, reinforcing the necessity for continued and robust clinical investigation.

Overall, the therapeutic landscape of ovarian cancer is gradually evolving through the integration of targeted, immunotherapeutic, and individualized strategies. The future of treatment lies in precision oncology, where molecular and immunologic profiling will inform clinical decisions and improve both survival outcomes and patient quality of life.

## Figures and Tables

**Table 1:** Bevacizumab-based phase III clinical trials in the first-line treatment of ovarian cancer.

Study	Treatment Strategy	Setting	No. of Patients	Primary Endpoint	Outcome	Adverse Events	Clinical Trial ID
GOG-0218 [27,28]	CTx (carboplatin AUC6 + paclitaxel 175 mg/m^2^, 6 cycles) • Control: + placebo (C2–22) • Bev-initiation: + bevacizumab 15 mg/kg (C2–6) → placebo (C7–22) • Bev-throughout: + bevacizumab 15 mg/kg (C2–22)	Newly diagnosed stage III (incompletely resectable) or stage IV EOC, FTC, or PPC	1873	PFS	PFS: 10.3 mo (control) vs. 11.2 mo (bev-initiation, HR = 0.91) vs. 14.1 mo (bev-throughout, HR = 0.72, *p* < 0.001) OS: No significant difference	HTN: 7.2% (control) vs. 16.5% (bev-initiation) vs. 22.9% (bev-throughout) GI perf: 1.2% vs. 2.8% vs 2.6%	NCT00262847
ICON7 [29,30]	CTx (carboplatin AUC5–6 + paclitaxel 175 mg/m^2^, 6 cycles) • Control: CTx only • Bev arm: + bevacizumab 7.5 mg/kg (C1–5/6) → maintenance bev (up to C18 or until PD)	First-line (incl. high-risk early & advanced disease)	1528	PFS	• PFS: 22.4 (control) → 24.1 mo (bev arm) (HR = 0.87, *p* = 0.04) • OS overall: No difference (44.6 vs 45.5 mo, *p* = 0.85)	↑ HTN (18% vs 2%), rare GI events with bev	NCT00483782
AGO-OVAR 17/BOOST [32]	Bev 15 mo vs. 30 mo + CTx (carboplatin AUC5 + paclitaxel 175 mg/m^2^ × 6)	First-line, FIGO stage IIb–IV EOC, FTC, or PPC	927	PFS	PFS & OS: No significant benefit with Bev 30 vs. Bev15	AEs ≥ G3 (Bev15 vs. Bev30): HTN 2.7% vs. 4.5%, thrombosis 2.2% vs 3.2%, fistula 3.1% vs. 1.1%, GI perf 0.2% vs. 0.9%, proteinuria 0.7% vs. 1.4%, hemorrhage 0.2% vs. 0.9%, MI 0% vs. 1.1%.	NCT01462890

Abbreviations: CTx: Chemotherapy, Bev: Bevacizumab, PFS: Progression-Free Survival, OS: Overall Survival, HR: Hazard Ratio, mo: Months, HTN: Hypertension, GI: Gastrointestinal, perf: Perforation, MI: Myocardial Infarction, AE(s): Adverse Event(s), ≥G3: Grade 3 or higher, EOC: Epithelial Ovarian Cancer, FTC: Fallopian Tube Cancer, PPC: Primary Peritoneal Cancer, FIGO: International Federation of Gynecology and Obstetrics, PD: Progressive Disease.

**Table 2:** Phase III trials evaluating PARP inhibitors as maintenance therapy in first-line advanced ovarian cancer.

Study	Treatment Strategy	Setting	No. of Patients	Primary Endpoint	Outcome	Adverse Events	Clinical Trial ID
SOLO1/GOG-3004 [37,38,39]	Maintenance olaparib 300 mg BID, up to 2 yrs vs. placebo after response to 1L platinum-based CTx	Newly diagnosed FIGO stage III–IV high-grade serous or endometrioid EOC/FTC/PPC with BRCA1/2 mutation	391	PFS	Median PFS: 56 mo (olaparib) vs. 13.8 mo (placebo); HR = 0.33 7-yr OS: 67.0% (olaparib) vs. 46.5% (placebo); HR = 0.55	Most common grade ≥ 3: anemia (21.9%) MDS/AML: low incidence	NCT01844986
PRIMA/ENGOT-OV26/GOG-3012 [40,41]	Maintenance niraparib QD up to 3 yrs vs. placebo after response to 1L platinum-based CTx	Newly diagnosed FIGO stage III–IV high-grade serous or endometrioid EOC/FTC/PPC	733	PFS (HRD & overall population)	HRD: median PFS 21.9 mo (niraparib) vs. 10.4 mo (placebo); HR = 0.43 Overall: median PFS 13.8 mo (niraparib) vs. 8.2 mo (placebo); HR = 0.62 Final OS: NS; (HR = 1.01)	Most common grade ≥ 3: anemia (31%), thrombocytopenia (28.7%), neutropenia (12.8%) MDS/AML: <2.5%	NCT02655016
ATHENA-MONO/GOG-3020/ENGOT-ov45 [42,43]	Rucaparib BID up to 2 yrs vs. placebo after response to 1 L platinum-based CTx	Newly diagnosed FIGO III–IV HGSOC	538	PFS (HRD, ITT)	HRD: PFS 28.7 mo (rucaparib)vs. 11.3 mo (placebo); HR = 0.47 ITT: PFS 20.2 mo (rucaparib) vs. 9.2 mo (placebo); HR = 0.52	Most common grade ≥ 3: anemia (28.7%), neutropenia (14.6%), ↑ALT/AST (10.6%), thrombocytopenia (7.3%)	NCT03522246
VELIA/GOG-3005 [44]	Control: Carboplatin + Paclitaxel + Placebo (6 cycles) → Placebo maintenance Veliparib combination only: Carboplatin + Paclitaxel + Veliparib (6 cycles) → Placebo maintenance Veliparib throughout: Carboplatin + Paclitaxel + Veliparib (6 cycles) → Veliparib maintenance (400 mg BID, up to 30 cycles)	Newly diagnosed FIGO stage III–IV HGSOC	1140	PFS (BRCA, HRD, ITT)	BRCA-mut: PFS 34.7 mo (veliparib-throughout) vs. 22.0 mo (control); HR = 0.44 HRD: PFS 31.9 mo (veliparib-throughout) vs. 20.5 mo (control); HR = 0.57 ITT: PFS 23.5 mo (veliparib-throughout) vs. 17.3 mo (control); HR = 0.68	Anemia, thrombocytopenia more frequent with veliparib + chemo	NCT02470585
PAOLA-1/ENGOT-ov25 [45,46]	Control: Bev (15 mg/kg q3w for up to 15 mo) + Placebo maintenance (24 mo) Experimental: Bev (15 mg/kg q3w for up to 15 mo) + Olaparib (300 mg BID, up to 24 mo)	Newly diagnosed FIGO stage III–IV HGSOC in response after platinum-based CTx + bevacizumab	806	PFS	HRD+: PFS 37.2 mo (olaparib + bev) vs. 17.7 mo (control); HR = 0.33 HRD+/BRCAwt: PFS 28.1 mo vs. 16.6 mo; HR = 0.43 ITT: PFS 22.1 mo vs. 16.6 mo; HR = 0.59 OS (ITT): 56.5 vs. 51.6 mo; HR = 0.92; NS HRD+: OS 5-yr OS 65.5% vs. 48.4%; HR = 0.62	AE(s) profile consistent with olaparib + bev	NCT02477644

Abbreviations: CTx: Chemotherapy, Bev: Bevacizumab, PFS: Progression-Free Survival, OS: Overall Survival, HR: Hazard Ratio, mo: Months, AE(s): Adverse Event(s), EOC: Epithelial Ovarian Cancer, FTC: Fallopian Tube Cancer, PPC: Primary Peritoneal Cancer, FIGO: International Federation of Gynecology and Obstetrics, BID: Twice Daily, QD: Once Daily, q3w: Every 3 Weeks, HRD: Homologous Recombination Deficiency, BRCAwt: BRCA wild-type, HGSOC: High-Grade Serous Ovarian Cancer, MDS: Myelodysplastic Syndrome, AML: Acute Myeloid Leukemia, ITT: Intention-To-Treat, ALT/AST: Alanine/Aspartate Aminotransferase, yrs: Year(s), NS: Not Significant.

**Table 3:** Immune checkpoint inhibitors phase III clinical trials in ovarian cancer.

Study	Treatment Strategy	Setting	No. of Patients	PD-L1 Selection	Primary Endpoint	Outcome	Clinical Trial ID
JAVELIN-100 [70]	Avelumab + CTx followed by avelumab maintenance vs CTx followed by avelumab maintenance vs. CTx alone	First-line	998	No	PFS	Negative (futility analysis)	NCT02718417
JAVELIN-200 [71]	Avelumab monotherapy or avelumab + PLD vs. PLD alone	Platinum-resistant recurrent	566	No	PFS, OS	Negative	NCT02580058
IMagyn050 [72]	Atezolizumab + CTx (carboplatin-paclitaxel) + bevacizumab vs. placebo	First-line	1301	Yes (stratified)	PFS, OS	Negative	NCT03038100
ATALANTE/ENGOT-ov29 [73]	Atezolizumab + CTx + bevacizumab vs. placebo	Platinum-sensitive recurrent	614	Yes (38% PD-L1+)	PFS	Negative	NCT02891824
NRG-GY009 [74]	Atezolizumab + PLD + bevacizumab vs PLD + bevacizumab vs. PLD + atezolizumab	Platinum-resistant recurrent	444	Exploratory (PD-L1, TILs)	PFS, OS	Negative	NCT02839707
AGO-OVAR 2.29 [75]	Atezolizumab + non-platinum CTx (weekly paclitaxel or PLD) + bevacizumab vs. placebo	Platinum-resistant recurrent	574	Yes (mandatory PD-L1)	PFS, OS	Negative	NCT03353831
ANITA (ENGOT-OV41) [76]	Atezolizumab + platinum CTx followed by niraparib maintenance vs. placebo	Platinum-sensitive recurrent	417	Yes (36% PD-L1+)	PFS	Negative	NCT03598270
DUO-O [77]	Durvalumab + platinum-based CTx + bevacizumab, followed by durvalumab + bevacizumab + olaparib maintenance vs. platinum-based CTx + bevacizumab followed by bevacizumab	First-line (non-BRCA)	1130	No	PFS	Positive (PFS)	NCT03737643
ATHENA Combo [78]	Nivolumab + rucaparib vs. rucaparib monotherapy	First-line maintenance	863	No	PFS	Negative	NCT03522246
FIRST (ENGOT-OV44) [79]	CTx (carboplatin-paclitaxel) ± bevacizumab followed by placebo maintenance vs. CTx (carboplatin-paclitaxel) ± bevacizumab followed by niraparib maintenance vs. Dostarlimab + CTx (carboplatin-paclitaxel) ± bevacizumab followed by niraparib + dostarlimab maintenance	First-line	~1000	Yes (PD-L1 tested)	PFS	Ongoing	NCT03602859
KEYLYNK-001 [80,81]	Pembrolizumab + CTx (carboplatin-paclitaxel) ± bevacizumab → pembrolizumab + olaparib maintenance vs. CTx alone vs pembrolizumab alone maintenance	First-line (BRCA-wt)	1367	Yes (CPS ≥ 10)	PFS	Positive pembrolizumab/olaparib vs CTx; pembrolizumab alone negative in CPS ≥ 10; intriguing benefit without bevacizumab in exploratory analyses	NCT03740165
KEYNOTE-B96 [82]	Pembrolizumab + weekly paclitaxel ± bevacizumab vs. placebo + weekly paclitaxel ± bevacizumab	Platinum-resistant recurrent	~616	Yes (CPS score)	PFS	Ongoing	NCT05116189

Abbreviations: PFS: Progression-Free Survival, OS: Overall Survival, PLD: Pegylated Liposomal Doxorubicin, CTx: Chemotherapy, TILs: Tumor-Infiltrating Lymphocytes, PD-L1: Programmed Death-Ligand 1, CPS: Combined Positive Score, BRCA-wt: BRCA Wild-Type, wt: Wild-Type.

## Data Availability

Not applicable.

## Abbreviations

The following abbreviations are used in this manuscript:

CRS	Cytoreductive Surgery
CC	Completeness of Cytoreduction
PARP	Poly-ADP Ribose Polymerase
NACT	Neoadjuvant Chemotherapy
VEGF	Vascular Endothelial Growth Factor
PFS	Progression Free Survival
OS	Overall Survival
EMA	European Medicines Agency
FDA	Food and Drug Administration
HR	Homologous Recombination
SSBs	Single-Strand Breaks
DSBs	Double-Strand Breaks
HRD	Homologous Recombination Deficiency
HGSOG	High-Grade Serous Ovarian Cancer
PFI	Progression Free Survival
PLD	Pegylated Liposomal Doxorubicin
FRa	Folate Receptor a
ADC	Antibody-Drug Complex
DOR	Duration Of Response
ORR	Objective Response Rate
DC	Dendritic Cell
ACT	Adoptive Cell Therapy
NK	Natural Killer
ADCs	Antibody-Drug Conjugates
TF	Tissue Factor
